# Deciphering the Role of Putative Novel miRNAs Encoded From the Newly Found Genomic Regions of T2T‐CHM13 in the Progression of Collecting Duct Renal Cell Carcinoma

**DOI:** 10.1002/cam4.70925

**Published:** 2025-04-30

**Authors:** Anik Mojumder, Sazzad Shahrear, Abul Bashar Mir Md. Khademul Islam

**Affiliations:** ^1^ Department of Genetic Engineering and Biotechnology University of Dhaka Dhaka Bangladesh

**Keywords:** cancer, carcinoma, collecting duct, epigenetic modulator, miRNA, novel, T2T‐CHM13, tumor‐suppressor

## Abstract

**Background:**

Collecting duct renal cell carcinoma (cdRCC) is a rare and aggressive renal cancer subtype. The molecular mechanisms underlying cdRCC remain poorly understood, which presents a significant challenge for the development of effective treatment strategies. Advances in genome sequencing, particularly the discovery of new genomic regions in the T2T‐CHM13 reference genome, have provided an opportunity to expand our understanding of this disease.

**Aims:**

Our study specifically aims to investigate the role of miRNAs encoded by these regions, proposing them as potential epigenetic regulators in the pathogenesis of cdRCC.

**Methods:**

We used bioinformatic pipelines and small RNA‐seq data analysis to predict novel miRNAs from the newly discovered genomic regions of T2T‐CHM13. RNA‐seq analysis of cdRCC tumors was performed to identify differentially expressed genes, and target prediction tools were used to find miRNA‐mRNA interactions. Functional enrichment analyses were conducted to characterize the biological pathways affected.

**Results and Discussion:**

Using computational approaches, we predicted 156 novel miRNAs from T2T‐CHM13's newly resolved sequences. RNA‐seq and miRNA‐mRNA interaction analyses identified 345 downregulated genes targeted by novel miRNAs and 395 downregulated genes targeted by known miRNAs. A comprehensive functional enrichment analysis of these perturbed genes revealed distinctive pathways, including cGMP‐PKG signaling, calcium signaling, adipocytokine signaling, PPAR signaling, and apelin signaling, all of which are implicated in tumorigenesis. Furthermore, Gene Ontology analysis linked miRNA‐targeted genes to disrupted cell–cell junctions and adhesion, providing a mechanistic explanation for aggressive invasion and metastasis in cdRCC. Additionally, a significant number of the target genes involved in metabolic and ion transport pathways were perturbed, explaining metabolic alterations in the cancer cells. We also identified 15 tumor suppressor genes downregulated by novel miRNAs, 6 of which were uniquely targeted, highlighting the potential of these miRNAs as cdRCC‐specific biomarkers.

**Conclusion:**

In conclusion, our study offers valuable insights into cdRCC biology from an epigenetic perspective, laying the groundwork for future research aimed at developing targeted therapies.

## Graphical abstract

We identified 156 novel miRNAs through de novo prediction in the newly discovered genomic regions of T2T‐CHM13. These miRNAs target 345 protein‐coding genes that are downregulated in collecting duct renal cell carcinoma (cdRCC). The miRNAs may play a role in the disease by altering several key tumor suppressor pathways and targeting multiple tumor suppressor genes.

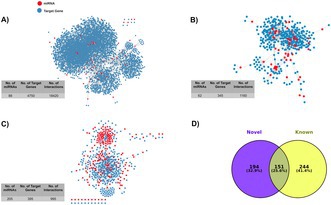

## 1 Introduction

Collecting duct renal cell carcinoma (cdRCC) is a rare but aggressive form of renal cell carcinoma (RCC), representing 1%–2% of all RCC cases [1, 2]. The most common symptoms include low back pain, hematuria, and fatigue [3]. cdRCC demonstrates a male predominance (male‐to‐female ratio 2:1) and typically presents at a median age of 59 years. Patients face a poor prognosis, with a median survival time of approximately 2 years [4, 5, 6]. As 42% of patients present with distant metastases at diagnosis, it is clear that early detection remains a challenge [4]. The molecular biology of cdRCC remains poorly understood, largely due to its rarity and lack of reports. However, several studies have contributed significantly to understanding its molecular signature. A study by Malouf and colleagues [7], identified distal convoluted tubules as a potential cellular origin and revealed distinct transcriptomic signatures differentiating cdRCC from other renal cancer subtypes, including metabolic shifts in pyruvate metabolism, tricarboxylic acid cycle activity, and immunogenic responses. Despite the unique signature, unsupervised clustering showed that the cdRCC transcriptome is more similar to that of clear cell RCC than to that of upper tract urothelial carcinomas. Comparative analysis with urothelial carcinomas highlighted upregulation of *CDH6* and *POU3F3*, and downregulation of *GATA3*, *TP63*, *KRT17*, *KRT20*, *UPK2*, *UPK1A*, and *UPK3A* in cdRCC [7]. Another study reported somatic single‐nucleotide variants (SNVs) in key cancer‐associated genes (including *ATM*, *CREBBP*, *PRDM1*, *CBFB*, *FBXW7*, *IKZF1*, *KDR*, *KRAS*, *NACA*, *NF2*, *NUP98*, *SS18*, *TP53*, and *ZNF521*), and identified associations between *CDKN2A* deletion, upregulation of SLC family genes (e.g., *SLC7A11*), and poor prognosis [8]. Cytogenetic studies further linked cdRCC with frequent DNA loss in chromosomes 1q, 6p, 8p, 9p, 21q, and chromosome Y with cdRCC [9]. However, studying the molecular basis from an epigenetic perspective could enhance our understanding of the disease.

miRNAs work as epigenetic regulators by targeting mRNAs and modulating the expression of the corresponding genes. Dysregulation of miRNAs has been found to be strongly correlated with the formation of cancers. For many RCC subtypes, it is well established through previous studies that miRNAs play a crucial role in their development [10]. These findings are encouraging research towards using these miRNAs as diagnostic and prognostic biomarkers [10, 11, 12, 13, 14]. Therefore, investigating the role of miRNAs in cdRCC can enable us to understand beyond our current knowledge of the biology of the disease. However, there has not been much miRNA‐based research on this rare RCC subtype. One comprehensive study to understand the correlation between known miRNAs and top deregulated genes in cdRCC was done by Wach et al. [15]. The strongest correlation was found for miR‐374b‐5p and miR‐26b‐5p with their target genes. Downregulation of miR‐374b‐5p was correlated with upregulation of its target genes such as *SLC7A11*, *HIST1H3B*, and *HK2*. On the other hand, the upregulation of miR‐26b‐5p was correlated with the downregulation of its target genes, including *PPARGC1A*, *ALDH6A1*, and *MARC2*. Expression levels of *HK2*, *PPARGC1A*, *ALDH6A1*, and *MARC2* were validated using cdRCC samples through qRT‐PCR [15]. However, detailed pathway analysis of the target genes of the known miRNAs, as well as novel miRNAs, is still needed.

With the release of the T2T‐CHM13 reference genome, including the gapless assemblies of all chromosomes (except Y) by the T2T consortium, errors of prior references are corrected, and about 200 million base pairs have been introduced [16]. T2T‐CHM13 contains both euchromatic and heterochromatic fractions of the genome, providing scope for functional, structural, and variational studies [16, 17, 18]. The currently used GRCh38 reference genome contains many errors and unsolvable gaps due to the limitations of bacterial artificial chromosome (BAC) cloning, the technology that was used to construct the assembly [16]. In contrast, PacBio HiFi and Oxford Nanopore ultralong‐read sequencing technology used to construct T2T‐CHM13 opened up 8% of the genome whose sequences were previously unknown [16, 19]. These newly found genomic regions may encode miRNAs that are currently unknown. Analyzing the role of the novel miRNAs alongside previously known miRNAs can provide more insights into cdRCC.

In this study, we predicted novel miRNAs from the newly found genomic regions of T2T‐CHM13. Through comparative analysis of their target genes with those of known miRNAs, we aimed to understand how these novel miRNAs contribute to the progression of cdRCC from a molecular viewpoint and enhance our understanding of cdRCC biology. Furthermore, we identified novel miRNA‐targeted downregulated key tumor suppressor genes, providing potential avenues for the development of therapeutics and the discovery of prognostic biomarkers.

## 2 Materials and Methods

**FIGURE 1:**
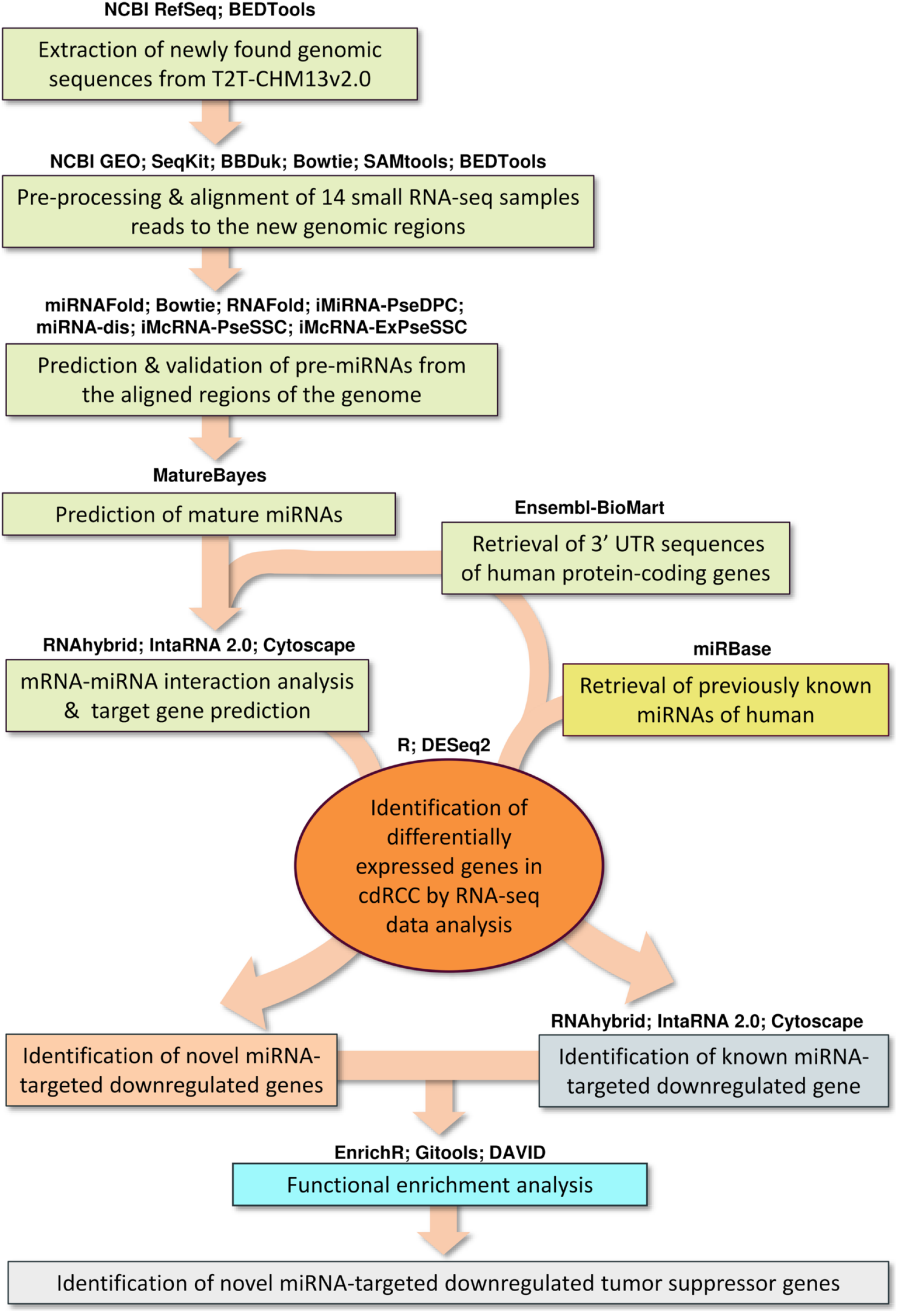
Workflow of identification of novel putative human miRNAs and understanding their role in cdRCC by analyzing their target genes.

A methodological overview is presented in Figure 1.

### 2.1 Obtaining Data

The reference genome of T2T‐CHM13v2.0 (Telomere‐to‐Telomere assembly of the CHM13 cell line, with chrY from NA24385) was obtained from the NCBI RefSeq database (RefSeq GCF_009914755.1) [20]. A PAF file (grch38‐chm13v2.paf) containing information on the alignment between GRCh38 and T2T‐CHM13v2 genomes was obtained from https://github.com/marbl/CHM13 [21]. For the prediction of miRNAs, 14 small RNA‐seq data samples were obtained from the GEO (Gene Expression Omnibus) database of NCBI (GEO accession: GSE198122) [20, 22]. These datasets contained samples from seven healthy male controls and seven male opioid use disorder patients [22]. The 3' UTR sequences of human protein‐coding genes were extracted using Ensembl‐BioMart (version 108) [23]. The RNA‐seq data count table matrix was obtained from a study on collecting duct renal cell carcinoma conducted by Wach et al., which contained count data of two carcinoma cases and eight normal tissue samples histologically [15]. The FASTA format sequences of all known mature miRNA sequences were retrieved from miRBase [24, 25].

### 2.2 Extracting Newly Found Genomic Regions of T2T‐CHM13

From the grch38‐chm13v2.paf file and T2T‐CHM13v2.0 reference genome FASTA file, a BED file containing the coordinates of the T2T‐CHM13v2 genome that did not directly align to the GRCh38 reference genome was formed using the awk tool and BEDTools (v2.27.1) [26]. The BED file and the T2T‐CHM13v2 reference genome FASTA file were used to create a new FASTA file that contained the sequences of the newly discovered regions of the human genome. BEDTools (v2.27.1) *getfasta* was used in this step [26].

### 2.3 Prediction of Pre‐miRNAs

An in‐house automated shell script was prepared to align the small RNA‐seq reads (GEO accession: GSE198122) to the newly found genomic regions. In the automated script, SeqKit (v2.3.0) [27] was used to remove duplicate reads, and BBDuk [28] was used to trim adapters for each sample. In the next step of the automated script, the trimmed reads were aligned to the newly found genomic regions using Bowtie (v1.2.3), a very fast and memory‐efficient short‐read aligner [29]. During alignment, no multi‐mapping and mismatch were allowed. In the subsequent steps of this automation, conversion from SAM files to BAM files, sorting of the alignment data in each BAM file, and removal of duplicates were done using SAMtools (v1.10) [30]. The BAM files were merged and sorted using SAMtools (v1.10) [30]. The starting coordinates for each alignment were obtained, and extended sequences of 150 nucleotides both forward and backward were extracted using BEDTools (v2.27.1) [26].

miRNAFold, a tool for predicting miRNA precursors from the genome, was used for de novo prediction of pre‐miRNAs from the extended sequences [31]. Parameters were kept default in this case. Only the pre‐miRNAs where at least one trimmed read (length range between 16 and 28 nucleotides) aligns completely with zero mismatches were taken for downstream analysis. This was done using Bowtie (v1.2.3) [29]. Pre‐miRNAs having stable secondary structures were selected by using the RNAFold program of the Vienna package [32, 33]. Here, pre‐miRNAs with MFE < − 45 kcal/mol were kept because of their higher thermal stability. Furthermore, false pre‐miRNAs were excluded using four more tools, which are iMiRNA‐PseDPC [34], miRNA‐dis [35], iMcRNA‐PseSSC [36], and iMcRNA‐ExPseSSC [36]. After the exclusion of false pre‐miRNAs declared by the algorithms of the tools, the remaining pre‐miRNAs were used for downstream analysis.

### 2.4 Prediction of Mature miRNAs

From the pre‐miRNA sequences, mature miRNAs were predicted utilizing MatureBayes, where a Naive Bayes classifier algorithm was implemented and both sequence and secondary structure information of the pre‐miRNAs were taken into account [37].

### 2.5 cdRCC RNA‐Seq Data Analysis

After retrieving RNA‐seq raw read‐count data from the study conducted by Wach and colleagues [15], differential expression (DE) analysis was conducted using the Bioconductor package DESeq2 v1.36.0 with R version 4.2.2 [38]. This tool performs gene‐level differential expression analysis using a negative binomial distribution [38]. The transcripts having total reads of at least 15 were considered. Differentially expressed genes were filtered on the basis of satisfaction of the following condition:|log_2_(Fold change)| > 2 and adjusted *p*‐value < 0.05. Then, the downregulated transcripts were filtered out for downstream analysis.

### 2.6 Predicted miRNA and Human mRNA Interaction Analysis

The interactions between the miRNAs and human mRNAs were studied with a view to predicting the target genes of the miRNAs. RNAhybrid [39] and IntaRNA 2.0 [40] were employed to assess the interaction between the novel miRNAs and 3' UTR sequences of human protein‐coding genes. In the case of RNAhybrid, only the mRNAs that interacted with the miRNAs with MFE ≤ − 30 kcal/mol and *p*‐value < 0.001 were considered. While using IntaRNA 2.0, the parameters used were–mode = H, −model = X, and–outMode = C. Only the mRNAs having an interaction energy of less than − 30 kcal/mol with the miRNAs were considered. The common predictions of these tools were taken into account for further analysis. Among the target genes, only the genes that were found to be downregulated in cdRCC were retrieved. Again, using the same tools and parameters, potential target genes of known miRNAs that are downregulated in cdRCC are identified. Network diagrams of the interactions between miRNAs and their target genes were created using Cytoscape v3.9.1 [41]. A Venn diagram of the overlap between the novel miRNA target genes and previously known miRNA target genes that are downregulated in cdRCC was created using Venny 2.1 [42].

### 2.7 Functional Enrichment Analysis of the Target Genes

The target genes of the novel predicted miRNAs that were found to be downregulated in cdRCC were used for functional enrichment analyses using EnrichR [43]. Other enrichment analysis tools, including Gitools [44] and DAVID 6.8 [45] were used in addition to EnrichR [43]. The downregulated target genes of known miRNAs were also subjected to functional enrichment analysis using the same tools. Moreover, the downregulated target genes of novel and known miRNAs were merged and were also used for the enrichment analysis. Gitools was used to create the heatmaps in order to compare the three gene sets for enrichment analyses [44].

## 3 Results

### 3.1 Extraction of the Newly Found Genomic Regions

From the reference genome sequence of T2T‐CHM13v2.0 (Telomere‐to‐Telomere assembly of the CHM13 cell line, with chrY from NA24385) [20], the sequences of the newly found genomic regions that do not directly align to the GRCh38 reference genome were extracted and found to be about 251 million nucleotides long.

### 3.2 Prediction of Novel miRNAs Encoded From the Newly Found Genomic Regions

The genomic regions were further narrowed down based on the alignment of the trimmed reads of small RNA‐seq data (GEO accession: GSE198122) [22]. Inside those extended aligned regions, miRNAfold was used to predict miRNA hairpin structures. Only the predicted pre‐miRNAs having at least one trimmed small RNA‐seq read aligned completely with it were taken into account. Further use of validation tools, including RNAFold, miRNA‐PseDPC, miRNA‐dis, iMcRNA‐PseSSC, and iMcRNA‐ExPseSSC, provided 78 pre‐miRNAs [32, 33, 34, 36]. Any pre‐miRNA not fulfilling the criteria of true pre‐miRNA by any of the mentioned algorithms was removed. By the use of the MatureBayes tool, 156 mature miRNAs were obtained from the 78 pre‐miRNAs [37] (Data S1).

### 3.3 Gene Expression RNA‐Seq Data Analysis of Collecting Duct Renal Cell Carcinomas (cdRCC)

**FIGURE 2:**
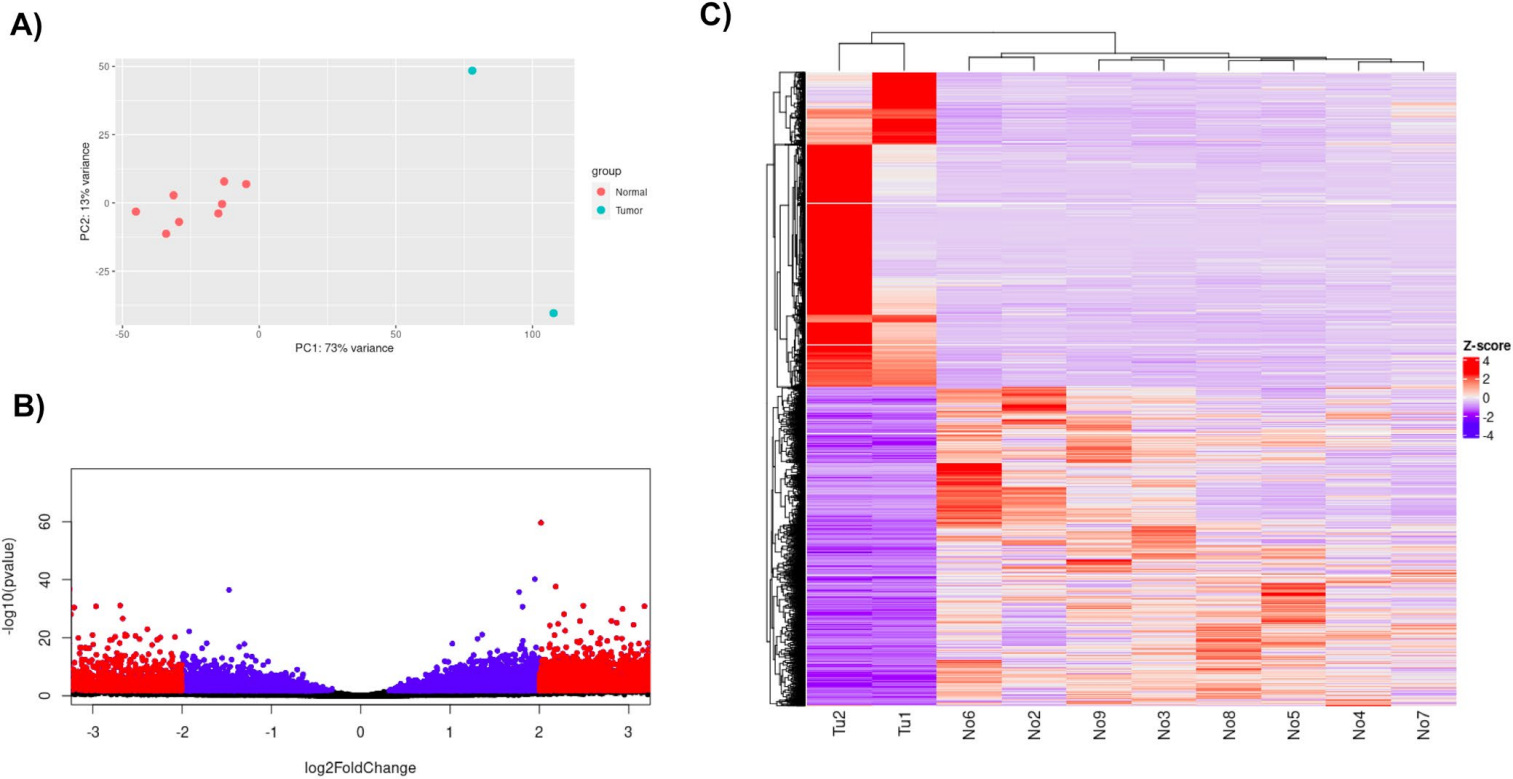
RNA‐seq analysis of collecting duct renal cell carcinoma (cdRCC) versus normal samples. (A) Principal component analysis (PCA) plot of cdRCC versus normal samples, showing a clear separation between the two groups. (B) Volcano plot of differentially expressed genes between cdRCC and normal samples, with differentially expressed genes in red (adjusted *p*‐value < 0.05 and |log2FoldChange| > 2) and non‐differentially expressed genes in blue (adjusted *p*‐value < 0.05 and |log2FoldChange| ≤ 2) and black (adjusted *p*‐value ≥ 0.05 and |log2FoldChange| ≤ 2). Genes represented in red with a positive log2FoldChange are upregulated, while those with a negative log2FoldChange are downregulated. (C) Heatmap of the differentially expressed genes between cdRCC and normal samples showing the relative expression levels of these genes across all samples, with red indicating high expression and blue indicating low expression. The abbreviation “Tu” represents tumor samples, while “No” represents normal samples.

The RNA‐seq count table data prepared by Wach et al. [46] was used for differential gene expression analysis. Principal Component Analysis between cdRCC samples and normal samples showed a clear separation between the groups (Figure 2A). Although the 2 cdRCC samples are distant from each other along the principal component 2 (PC2) axis, they are near to each other along the principal component 1 (PC1) axis. The result showing that PC1 accounts for 73% variance and PC2 accounts for 13% of the total variance between the samples helps conclude that the two cdRCC samples are more similar to each other than other normal samples due to their closeness along the PC1 axis. Using thresholds of |log_2_(Fold change)| > 2 and adjusted *p*‐value < 0.05, 4839 genes were found to be differentially expressed. Among them, 2637 genes were upregulated and 2202 genes were downregulated (Figure 2B,C; Tables S1–S18).

### 3.4 Identification of Putative Downregulated Target Genes of Novel miRNAs and Previously Known miRNAs

**FIGURE 3:**
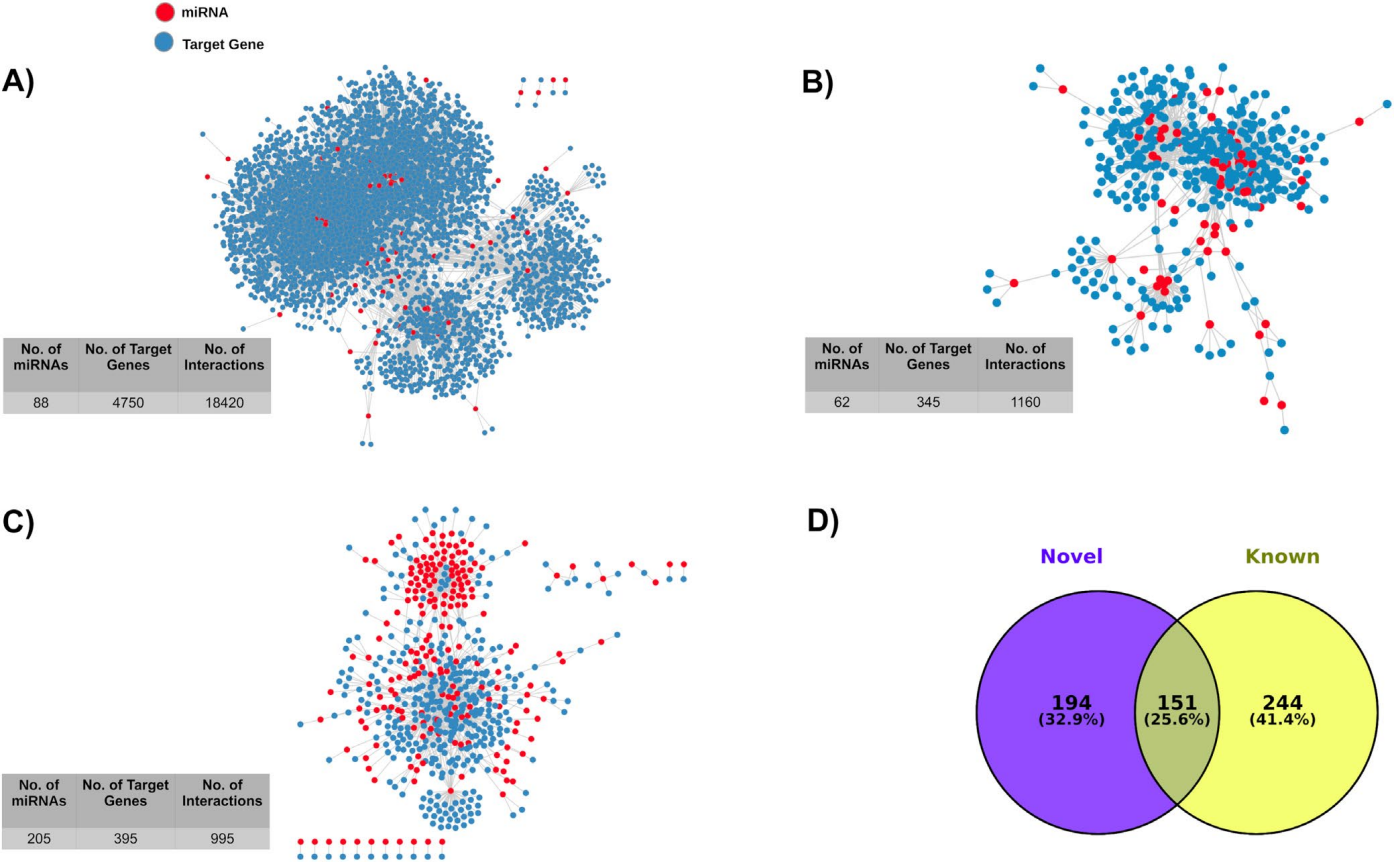
Network analysis of miRNA‐target gene interactions and identification of downregulated target genes in collecting duct renal cell carcinoma (cdRCC). (A) Network diagram of the interactions between novel miRNAs and their target genes. (B) Network diagram of the interactions between novel miRNAs and their target genes that are downregulated in cdRCC. (C) Network diagram of the interactions between previously known miRNAs and their target genes that are downregulated in cdRCC. (D) Venn diagram of the overlap between the novel miRNA target genes and previously known miRNA target genes that are downregulated in cdRCC.

The predicted novel miRNAs of newly found human genomic regions might play an important role in various types of pathways by targeting specific mRNAs. We performed *in silico* predictions of identifying the target genes with a very stringent cutoff using RNAHybrid and IntaRNA 2.0 to reduce false positives [39, 40]. Among the 156 novel miRNAs, 88 miRNAs were found to target 4750 genes (Figure 3A; Table S2). Very stringent cutoffs might be the reason for not getting any targets for the rest of the novel miRNAs. Among the targets, 345 genes were found to be downregulated in cdRCC, which were targeted by 62 novel miRNAs, indicating their potential role in the disease (Figure 3B; Table S3). Using the same cutoffs, the target genes of previously known 2656 miRNAs that were found to be downregulated in cdRCC were identified. Here, 395 downregulated genes were found to be targeted by 205 previously known miRNAs (Figure 3C; Table S4). Among the downregulated genes, 194 genes were uniquely targeted by novel miRNAs, while 244 genes were uniquely targeted by the previously known miRNAs. 151 genes were targeted by both the novel and previously known miRNAs (Figure 3D; Tables S5 and S6).

### 3.5 Functional Enrichment Analysis of the Target Downregulated Genes

**FIGURE 4:**
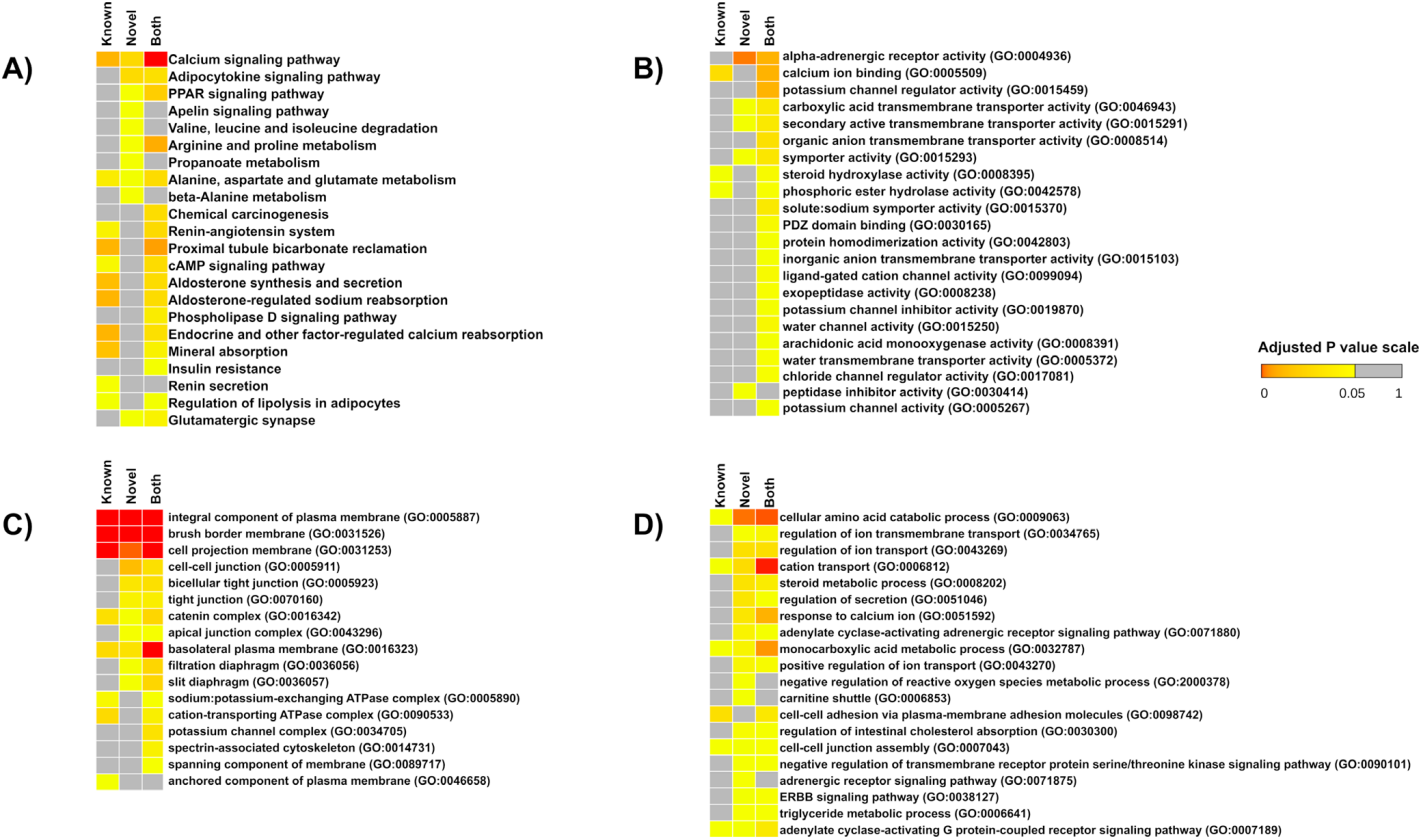
Results of enrichment analyses of miRNA target genes that are downregulated in cdRCC. Each heatmap in the figure represents the results of enrichment analyses for miRNA target downregulated genes in cdRCC including selected, (A) KEGG pathways, (B) GOMF terms, (C) GOCC terms, and (D) GOBP terms. Each heatmap is divided into three columns, namely “Novel”, representing novel miRNA target genes, “Known”, representing previously known miRNA target genes, and “Both”, representing both miRNA target genes. The color intensity in the heatmaps indicates the significance level, with colors tending towards red indicating more significance, yellow indicating less significance, and gray indicating non‐significant enrichment.

Functional annotation and enrichment analyses were performed to understand the biological role and pathways conferred by the target genes of novel miRNAs. Here, we performed a comparative analysis of enrichment results between novel miRNA target genes, previously known miRNA target genes, and the combination of the two sets of genes that were downregulated in cdRCC (Figure 4). This was done in order to understand how the novel miRNAs add to the knowledge of cdRCC biology.

Analyzing the KEGG (Kyoto Encyclopedia of Genes and Genomes) pathways enriched for the novel miRNA targets, we found that the cGMP‐PKG signaling pathway, calcium signaling pathway, adipocytokine signaling pathway, PPAR signaling pathway, and apelin signaling pathway are downregulated in cdRCC (Figure 4A; Tables S7–S9). This perturbation might be directly involved in carcinogenesis. There is much evidence for the role of their dysregulation in cancer. Here, the cGMP‐PKG signaling pathway and calcium signaling pathway are enriched for both known miRNA and novel miRNA targets. However, the adipocytokine signaling pathway, PPAR signaling pathway, and apelin signaling pathway are only overrepresented for the novel miRNA targets. These indicate the mechanism by which the novel miRNAs might play a role in the formation and progression of cdRCC. The targeted genes in the signaling pathways have been marked in their pathway maps (Figure S1).

Gene Ontology Molecular Function (GOMF) analysis (Figure 4B; Tables S10–S12) suggests the following terms enriched for novel miRNA targets downregulated in cdRCC: alpha‐adrenergic receptor activity, carboxylic acid transmembrane transporter activity, peptidase inhibitor activity, secondary active transmembrane transporter activity, and symporter activity.

Gene Ontology Cellular Component (GOCC) analysis (Figure 4C; Tables S13–S15) indicates that target genes downregulated in cdRCC are responsible for forming some crucial subcellular structures that are associated with the plasma membrane. They include tight junction and bicellular tight junction, cell–cell junction, catenin complex, brush border membrane, cell projection membrane, apical junction complex, filtration diaphragm, and slit diaphragm. Among these, the GO terms brush border membrane, catenin complex, and cell projection membrane are found to be enriched in the case of both known and novel miRNA targets. The rest of the terms were enriched uniquely in the case of novel miRNA targets. Dysregulation of the mentioned components might be associated with tumorigenesis. Deregulation of the filtration diaphragm and slit diaphragm might be a direct indication of diseases and malfunction of kidneys.

Gene Ontology Biological Process (GOBP) analysis (Figure 4D; Tables S16–S18) shows an association of downregulation of several enriched terms in cdRCC. Among them are ion transport, especially sodium and potassium, secretion, response to calcium ions, reactive oxygen species metabolic process, carnitine shuttle, cell–cell junction assembly, and cell‐substrate adhesion. miRNAs responsible for dysregulating them can also play an important role in the tumorigenesis of cdRCC.

The gene ontology studies indicate that the novel miRNA‐targeted downregulated genes are mostly responsible for ion transport and cellular adhesion. Disruption of cellular adhesion and junction due to downregulation of the target genes might be correlated with enhanced migration and invasion of the tumor cells. Disruption in the ions and solute transmembrane transport activity might be linked to altered signaling in the tumor microenvironment. This altered signaling, along with an altered response to signaling, might be correlated with the uncontrolled proliferation, growth, transformation, and invasion of the cdRCC cells.

### 3.6 Identification of Downregulated Tumor Suppressor Genes Targeted by Novel miRNAs

**FIGURE 5:**
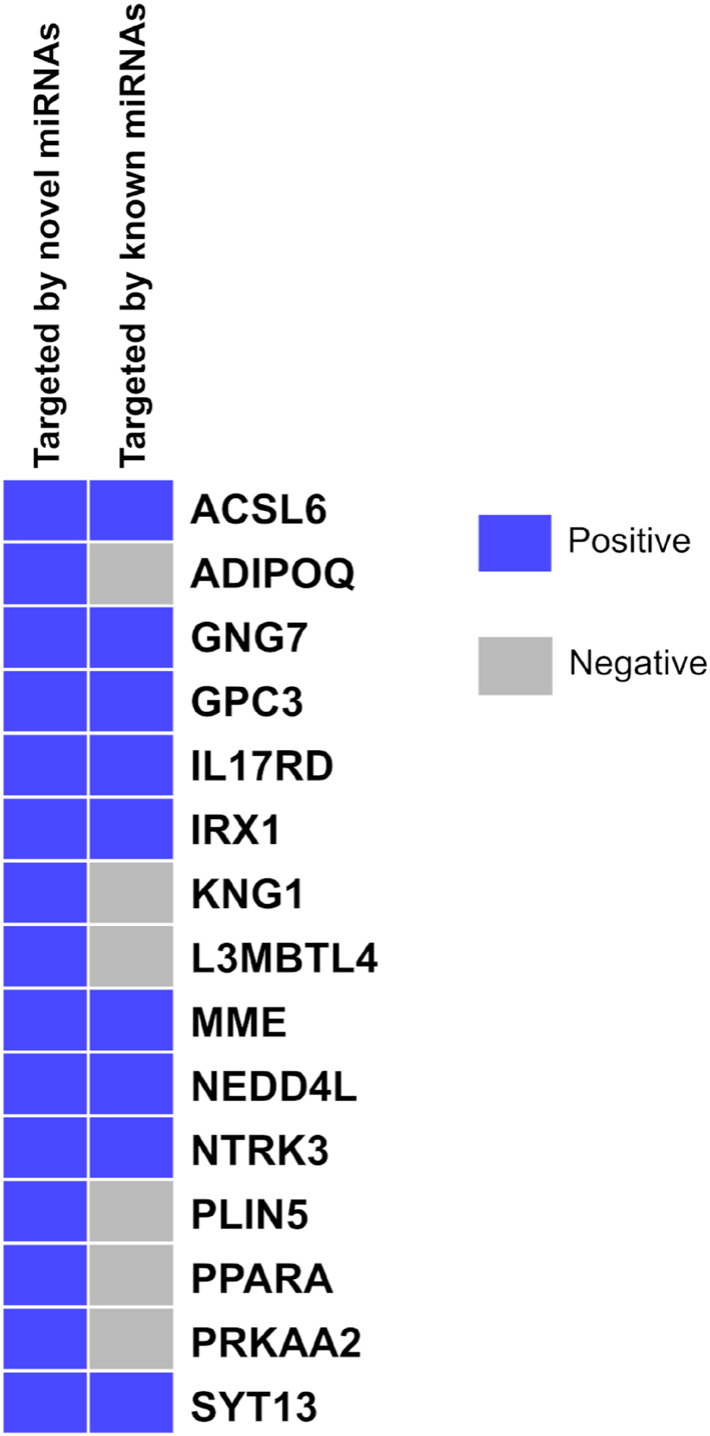
A heatmap of selected downregulated tumor suppressor genes in cdRCC, indicating whether they are targeted by novel or previously known miRNAs. Blue cells indicate targeting by the corresponding miRNA set, while gray cells indicate non‐targeting.

Among the novel miRNA‐targeted downregulated genes in cdRCC, we have identified 15 tumor suppressor genes. They are *PRKAA2* [47], *ADIPOQ* [48], *PPARA* [49], *ACSL6* [50], *PLIN5* [51], *GNG7* [52], *KNG1* [53], *NTRK3* [54], *NEDD4L* [55], *GPC3* [56], *MME* [57], *IL17RD* [58], *SYT13* [59], *IRX1* [60] and *L3MBTL4* [61]. Among them, *ADIPOQ*, *KNG1*, *L3MBTL4*, *PLIN5*, *PPARA*, and *PRKAA2* are uniquely targeted by the novel miRNAs (Figure 5).

## 4 Discussion

As described earlier, the newly found regions of the T2T‐CHM13 reference genome could serve as an important source of encoded cellular miRNAs that regulate gene expression. These miRNAs represent promising therapeutic targets, and their discovery could enable novel therapeutics.

Predicting the pre‐miRNAs was a crucial step in our workflow. We focused on genomic regions where trimmed reads from multiple small RNA‐seq datasets aligned without mismatches. The use of five independent tools improved prediction accuracy, and selecting pre‐miRNAs based on perfect alignment with small RNA‐seq reads served as in silico validation. Finally, using the MatureBayes tool [37], which employs a Naive Bayes classifier algorithm, we identified 156 miRNAs with potential roles in epigenetic regulation, disease biology, and drug development. Here, we investigated their therapeutic potential in cdRCC by understanding their role.

We analyzed downregulated genes in cdRCC RNA‐Seq data [15] targeted by novel miRNAs and compared them to targets of known miRNAs from miRBase [25]. Enrichment analysis revealed individual and synergistic roles of novel/known miRNAs in cdRCC progression, uncovering potential mechanisms mediated by these miRNAs. An important aspect of tumorigenesis is the downregulation of tumor suppressor pathways [62]. Three cancer‐associated pathways were found to be significantly downregulated by the novel miRNAs. Among these, while adipocytokine and PPAR signaling are established tumor‐suppressor pathways, apelin signaling typically promotes tumorigenesis.

Many of the adipocytokines, produced by adipocytes and other cell types [63], work as modulators in tumor formation [64]. Adiponectin, a key adipocytokine, plays a role as a tumor suppressor by phosphorylating AMPK, downregulating MAPK, JAK/STAT, mTOR, and Wnt/β‐catenin pathways [64, 65], inhibiting metastases, and reversing epithelial‐mesenchymal transition [66]. Antagonizing role of adiponectin against leptin helps to explain how a decrease in adiponectin/leptin ratio increases the risk of cancers such as colorectal, prostate, hepatic, endometrial, and breast [67, 68]. Our analysis identified *PRKAA2*, *ADIPOQ*, *SLC2A4*, *PPARA*, *ACACB*, and *G6PC1* as targets of the novel miRNAs in adipocytokine signaling (Figure S1A). These genes are mostly involved in adiponectin signaling rather than leptin signaling. Thus, suppressing this tumor‐suppressing activity (adiponectin‐induced AMPK activation) might be an important role of the novel miRNAs.

The PPAR signaling pathway plays important roles in tumor suppression and regulation of inflammation [69]. PPARα exerts its antitumoral effects by inhibiting angiogenesis, disrupting glucose and lipid balance by prioritizing fatty acid oxidation leading to ATP production inhibition, inducing apoptosis and ferroptosis, regulating DNA methylation, accumulating reactive oxygen species and mitochondrial damage, etc., in different types of tumors [49]. In fact, the interplay and crosstalk of the PPAR signaling pathway with cancer from the perspective of metabolism have been studied immensely and these studies are providing insights into the potential of this pathway as a valuable therapeutic target [70]. We found that the genes *ADIPOQ*, *ACSL6*, *ACADM*, *PPARA*, *CYP8B1*, and *PLIN5* were downregulated among the novel miRNA targets (Figure S1B). The genes, except for *PPARA*, work downstream of the PPARs in the PPAR signaling pathway. Here, the fact that *PPARA* encodes PPARα makes the miRNAs targeting this gene an important target for the development of therapeutics against cdRCC. Previously, D. Portius and colleagues [71] addressed the reciprocal regulation of miRNA expression by PPARs in order to speculate on the therapeutic potential of pharmacological agents targeting miRNAs in altering PPAR expression/activity.

Unlike other pathways mentioned previously, the apelin signaling pathway (apelin/APJ axis) is involved in the migration of cancer cells, neoangiogenesis, induction of metastases, tumor growth, and proliferation [72]. This apelin/APJ system enhanced migration in different types of cancer cells, including lung adenocarcinoma, ovarian cancer, gastric cancer, and oral squamous cell carcinoma [73]. Possible modulated pathways by apelin for cell migration are thought to be the PAK1/cofilin signaling pathway, MAPK/ERK, AMPK, PI3K/Akt, and PPAR pathways [73]. Proangiogenic activities of the apelinergic system were also observed in multiple in vitro and in vivo studies [74, 75, 76, 77]. Paradoxically, apelin‐13 caused inhibition of TGF‐β‐induced epithelial‐mesenchymal transition in primary human renal proximal tubular epithelial cells [74, 78]. Moreover, the anti‐apoptotic or protective role of apelin is also studied thoroughly, which was exerted mostly by decreasing caspase‐3 activity [73]. Although the genes in (*PRKAA2*, *GNG7*, *MYL3*, *NOS1*, *ADCY1, PLCB1*, *MYLK3*, and *MYLK4*) the apelin/APJ signaling axis are found to be downregulated and might not be considered responsible in cdRCC progression, downregulation of *GNG7* tells a different story due to its tumor‐suppressive activity (Figure S1C). This gene functions as part of the APJ‐coupled G protein, and its low expression leads to a reduced G1 phase, contributing to clear cell renal cell carcinoma [52].

Another two pathways (cGMP‐PKG signaling and calcium signaling) that are downregulated in cdRCC are found to be targeted by both novel and known miRNAs. The cGMP‐PKG signaling pathway is considered a tumor‐suppressive pathway. This pathway plays an important role in inducing apoptosis, inhibition of tumor growth, suppression of proliferation, and angiogenesis in multiple types of cancer [79, 80, 81, 82, 83]. For example, PDE5 inhibition activates PKG, which reduces cyclin D1 (a cell cycle promoter) and elevates p21 (a cell cycle inhibitor), leading to apoptosis and suppressed proliferation in renal cancer cells [84]. In colon cancer, PKG also dampens tumorigenesis by inhibiting β‐catenin/TCF and SOX9 signaling, thereby reducing angiogenesis and proliferation [85]. These findings strongly link cGMP‐PKG pathway downregulation to tumorigenesis. Downregulation of several key genes that are targeted by the novel miRNAs (*KCNMB3*, *KCNMB4*, *ADRA1D*, *ATP1B2*, *ADCY1*, *ADRA1B*, *ADRA2C*, *PLCB1*, *ADRA1A*, *MYLK3*, *KNG1*, *MYLK4*) of this pathway may account for cdRCC progression (Figure S1D).

Deregulation in the calcium signaling pathway has been linked to cancer hallmarks in many studies [86]. Both upregulation and downregulation of this pathway can disrupt the Ca^2+^ homeostasis in cells and contribute to transforming normal cells. In various tumor models, perturbation in Ca^2+^ homeostasis resulted in consequences like promotion of cell proliferation, invasion, tumor angiogenesis, cell migration, metastases, reduced apoptosis, alterations in cell adhesion, cellular responses to some stimuli, intracellular communication through gap junctions, response to ATP effects, altered oxidative response, etc. [86] ATP‐P2RX6 modulates the Ca^2+^‐mediated p‐ERK1/2/MMP9 signaling to enhance the migration and invasion of renal carcinoma cells [87]. There is a strong interconnection between the calcium signaling pathway and cGMP signaling that forms a closed loop in regulation [81, 88]. The fact that the novel miRNAs target genes such as *EGF*, *NTRK3*, *ADRA1D*, *AVPR1A*, *ADCY1*, *ADRA1B*, *ADRA1A*, *MYLK3*, *MYLK4*, *GNAL*, *PLCE1*, *NOS1*, *PLCB1*, and *FGF10* may disrupt the calcium homeostasis, leading to modified signaling in the tumor microenvironment (Figure S1E). Here, *MYLK3* and *MYLK4* genes serve as bridges between cGMP/PKG signaling, calcium signaling, and apelin signaling pathways.

The Gene Ontology analysis of the novel miRNA‐targeted downregulated genes suggests the downregulation of cellular junctions that link them to tissue invasion and metastasis, one of the hallmarks of cancer [62]. This might help explain the aggressive nature of cdRCC. Perturbations in the transport of ions and solutes, response to regulatory ions, carnitine shuttle, the metabolic process of reactive oxygen species, and others are indicative of altered signaling and metabolism in cdRCC cells.

Recessive loss of function of tumor suppressor genes is considered an important cause of tumorigenesis [62]. However, this loss can also occur at the epigenetic level by alterations in the miRNA level that target those genes. Thus, understanding the role of miRNAs in this loss of function of tumor suppressor genes is also of great significance. Analyzing novel miRNA‐targeted downregulated genes, we have identified 15 tumor suppressor genes, of which 6 are uniquely targeted by the novel miRNAs. Their role in cancer is summarized in Table 1.

**TABLE 1:** Novel miRNA‐targeted tumor suppressor genes downregulated in cdRCC and their role in cancer.

Gene name	Encoded protein name	Cancer	Role in cancer	Reference
*PRKAA2*	**5'‐AMP‐activated protein kinase catalytic subunit alpha‐2**	Multiple	Metabolic tumor suppressor	[47]
*ADIPOQ*	**Adiponectin**	Breast cancer	Tumor suppression by inducing autophagic cell death	[48]
*PPARA*	**Peroxisome proliferator‐activated receptor alpha**	Multiple	Multiple anti‐tumor effects (i.e., inhibiting angiogenesis)	[49]
*ACSL6*	**Long‐chain‐fatty‐acid—CoA ligase 6**	Leukemia	Potential tumor suppressor	[50]
*PLIN5*	**Perilipin‐5**	Ovarian cancer	Demethylation of the gene inhibited cell proliferation, migration, and invasion, and increased apoptosis.	[51]
*GNG7*	**Guanine nucleotide‐binding protein G(I)/G(S)/G(O) subunit gamma‐7**	Clear cell renal cell carcinoma	Tumor suppressor	[52]
*KNG1*	**Kininogen‐1**	Glioma	Inhibition of proliferation and angiogenesis, promotion of apoptosis	[53]
*NTRK3*	**NT‐3 growth factor receptor**	Colorectal Cancer	Conditional tumor suppressor that induces apoptosis if NT‐3 is absent	[54]
*NEDD4L*	**E3 ubiquitin‐protein ligase NEDD4‐like**	Multiple	Tumor suppressor	[55]
*GPC3*	**Glypican‐3**	Lung cancer	Promotion of apoptosis	[56]
*MME*	**Neprilysin**	Multiple	Downregulation promoted angiogenesis, cell survival and cell migration	[57]
*IL17RD*	**Interleukin‐17 receptor D**	Prostate and breast cancer	Inhibition of cell migration and invasion and tumor growth, reduction of EMT markers (Snail and Slug)	[58]
*SYT13*	**Synaptotagmin‐13**	Liver cancer	Tumor suppressor having a role in pathways of mesenchymal to epithelial transition	[59]
*IRX1*	**Iroquois‐class homeodomain protein IRX‐1**	Lung cancer	Epigenetically regulated tumor suppressor	[60]
*L3MBTL4*	**L3MBTL histone methyl‐lysine binding protein 4**	Breast cancer	Potential tumor suppressor	[61]

As a disease resistant to standard treatment modalities and with limited options for adjuvant therapies, novel strategies for the treatment of cdRCC are required [89]. The novel miRNAs that downregulate key tumor suppressor genes of the tumor‐suppressing pathways can be targeted for developing new therapeutics for the disease. This can be done by inhibiting these miRNAs with antisense oligonucleotides [90, 91] and/or miRNA sponges [92, 93]. Furthermore, analyzing the expression profiles of novel miRNAs in a large cohort of cdRCC samples might enable the identification of potential biomarkers for diagnosis and prognosis of this disease. This could provide valuable knowledge, as this disease has a very poor prognosis, with approximately 42% of patients presenting with metastatic disease at the time of their diagnosis [4]. In addition to their diagnostic potential, these miRNAs also open avenues for personalized therapeutic strategies. Stratifying patients based on miRNA expression profiles could help guide therapeutic decisions, such as targeting specific miRNAs in cases where critical tumor suppressors are silenced. To translate these findings into clinical applications, future directions should include experimental validation using qRT‐PCR or Northern blot in tumor samples, luciferase assays for direct targeting confirmation, and miRNA mimic or inhibitor studies. Detection in biofluids like serum or urine could also support non‐invasive biomarker development. Nonetheless, challenges such as the rarity of cdRCC, inter‐patient variability in miRNA expression, and delivery system limitations for miRNA therapeutics must be addressed. Interdisciplinary collaboration involving genomics, molecular biology, and clinical research will be critical to overcoming these obstacles and translating miRNA discoveries into effective diagnostic and therapeutic tools.

## 5 Conclusion

A clear understanding of the molecular mechanisms behind cdRCC is still lacking, making it an area of active research. With the discovery of new genomic regions from the newly released T2T‐CHM13 reference genome, we now have a valuable resource to better comprehend this type of disease. To the best of our knowledge, our research team was the first to conduct an *in silico* prediction of novel miRNAs from these genomic regions and investigate their role in cdRCC. From an epigenetic perspective, we have attempted to elucidate the role of these miRNAs, uncovering potential causes for the high aggressiveness of cdRCC. Additionally, our findings suggest that these miRNAs could serve as effective drug targets and biomarkers for diagnosis and prognosis. By targeting tumor‐suppressive pathways and regulating critical genes, these miRNAs may support both therapeutic intervention and personalized treatment strategies. Their expression profiles could guide patient stratification and inform decisions on miRNA‐targeted therapies. In conclusion, while further research is necessary to enhance our comprehension of the disease and the function of the newly discovered miRNAs, our study has provided a basis for new and promising research directions that may lead to improved therapies and diagnostics.

## Author Contributions

**Anik Mojumder:** data curation (lead), formal analysis (lead), investigation (equal), methodology (equal), resources (lead), writing – original draft (lead), writing – review and editing (equal). **Sazzad Shahrear:** formal analysis (supporting), methodology (equal), writing – review and editing (supporting). **Abul Bashar Mir Md. Khademul Islam:** conceptualization (lead), investigation (equal), methodology (equal), project administration (lead), supervision (lead), validation (lead), writing – original draft (supporting), writing – review and editing (equal).

## Conflicts of Interest

The authors declare no conflicts of interest.

## Supporting information


**Data S1.** A file containing the sequences of the predicted novel human miRNAs.

**Figure S1.** KEGG pathway maps highlighting novel miRNA target genes downregulated in cdRCC, including, (A) Adipocytokine signaling pathway, (B) PPAR signaling pathway, (C) Apelin signaling pathway, (D) cGMP‐PKG signaling pathway, and (E) calcium signaling pathway. In each pathway map, novel miRNA target genes downregulated in cdRCC are highlighted with a yellow background.

Table S1‐S18.

## Data Availability

All data are added in the table, figures, and Supplementary Files and Supplementary Tables. In this research work, publicly available, free online and offline software/tools were used. Necessary links and references of the software/tools are provided in the methods section.
