# Supplementary material for: Deciphering the Role of Putative Novel miRNAs Encoded From the Newly Found Genomic Regions of T2T‐CHM13 in the Progression of Collecting Duct Renal Cell Carcinoma
Source: Cancer Med. 2025 Apr 30;14(9):e70925. doi: 10.1002/cam4.70925 (PMC12042112; doi:10.1002/cam4.70925)
Supplement: Supplementary file 1 — Data S1. A file containing the sequences of the predicted novel human miRNAs. [file CAM4-14-e70925-s001.docx]

>miR-ex-1

CACCGCCCGUCGCUCCAUGGUG

>miR-ex-2

CUUCAGGGGCACGGGCGUGCCA

>miR-ex-3

AGCCGAGAUCGUGCCACUGCAC

>miR-ex-4

CACUGCACUCCAGCCUGGGCAA

>miR-ex-5

CGGGCGCAGGAGCGCCCGGGAG

>miR-ex-6

CCCGGGAGCGCUAGUCGGUGCG

>miR-ex-7

AGAUUCUCAUAAGGAGCAUGCA

>miR-ex-8

UUUGUGCUCCUAUGAGAAUCUA

>miR-ex-9

GGGCUGGUCUGUCAGCCUAGGA

>miR-ex-10

UCCAGGGCUCCUUACCAGAAAA

>miR-ex-11

CGAGAUCAGACGAGAUCGGGCG

>miR-ex-12

GAUCGGGCGCGUUCAGGGUGGU

>miR-ex-13

CCUACUCUGAUUGGACUUUGUU

>miR-ex-14

AAAUAAAGUCCAAUCAGAGAAG

>miR-ex-15

GGCGGUGCGCCGCGACCGGCUC

>miR-ex-16

GGCGGGGGACCGUCCCCGGACC

>miR-ex-17

CCUCCUCCCCGGAGGGGGCGGG

>miR-ex-18

GCGGGCUCCGGCGGGUGCGGGG

>miR-ex-19

CGGCGGCGGCGGCGGGCGGCGG

>miR-ex-20

GGCGGCGGAGGGGCCGCGGGCC

>miR-ex-21

CCCGCCCUACCCCCCCGGCCCC

>miR-ex-22

CGGGGAGCGGUCGGGCGGCGGC

>miR-ex-23

CCUCCUCCCCGCGCCCCCGCCC

>miR-ex-24

UGGGGGGUGCCGCGCGCGGGUC

>miR-ex-25

UGCCGCGCGCGGGUCGGGGGGC

>miR-ex-26

CGGGCGGGUCGCGCCGUCGGGC

>miR-ex-27

UCUCCCCCGCGGGGGCGCGCCG

>miR-ex-28

AAUCCCCCGCGAGGGGGGUCUC

>miR-ex-29

CGCCGGGCACGGCCCCGCUCGC

>miR-ex-30

CCGUCGCCUCUGCCGCGCCGCU

>miR-ex-31

UCGCGGCGGGUCUGGGGGUCUC

>miR-ex-32

CCCCGCCGCGCCGGCUCCCCGU

>miR-ex-33

CGGCGGUGGCGGCGGCGGCGGC

>miR-ex-34

CCGAGUGUUACAGCCCCCCCGG

>miR-ex-35

UCCGCCCCCCGGCCCCGCGUCC

>miR-ex-36

CGCGCGGGUCGGGGCGGCGGCG

>miR-ex-37

AGACAGCCCUCGCUGUCGCCUC

>miR-ex-38

GGUGCCGUCCCGCCGGCCCGUC

>miR-ex-39

UGCGGGCGGCCCCCCUCCGCGG

>miR-ex-40

GGGUUUGCGCGAGCGUCGGCUC

>miR-ex-41

CAGAGUCUGGCUCUGUCUCCCA

>miR-ex-42

CCUCCGGGUUCACGCCAUUCUC

>miR-ex-43

CGCGGCCAGGCUGGGCUGGAGC

>miR-ex-44

UUGCUCCCUGGCUCAAGAAUGC

>miR-ex-45

CACGACACCGUCACCGGUUCGG

>miR-ex-46

UCGGGGAAGUAGUCCUUGACCA

>miR-ex-47

GAGUGCAGUGGCGCGAUCUCGG

>miR-ex-48

CUCAGCCUCCCGAGUAGCUGGG

>miR-ex-49

CGCCUCCACAUGGCUGAGGUGU

>miR-ex-50

CACCCUGGCCUGUGGAGCCAGG

>miR-ex-51

UUGAGCCCGGGGUGGAGGUGGC

>miR-ex-52

ACAGAGCAGGACUGUGUCUCAG

>miR-ex-53

AGGCAGGAGAAUGGCGUGAACC

>miR-ex-54

CACUGCACUCGAGCCUGGGCUA

>miR-ex-55

UUUGGGAGGCCAAGGUGGGUGG

>miR-ex-56

UUCAAGACCAGCCUGGCCACGA

>miR-ex-57

CAGGCUGAAGUGCAGUGGCGUG

>miR-ex-58

CACGCCAUUCUCCUGCCUCAGC

>miR-ex-59

AGGCAGGAGAAUUGCUUGAACC

>miR-ex-60

UGAACCCGGGAGGAAGAGGCUG

>miR-ex-61

CCUCCCAAAGUGUUGGGAUUAC

>miR-ex-62

UGGCCAGAGGCAAGGAGUUUGA

>miR-ex-63

CGGGCGGCGGGCGGCGAGCGGC

>miR-ex-64

CUCGCUCGGCUGCGGCUCCCGG

>miR-ex-65

UUUAAGGAGGGACUCACUCACA

>miR-ex-66

GGAGUAGGUGCCUCCUUAAAUU

>miR-ex-67

UCGACAGAGCAUGACCCUCUUU

>miR-ex-68

UGGAGUCUAGCUCUGUCGCCCA

>miR-ex-69

AACUGGCCAGUGAGCAGGAAGG

>miR-ex-70

UCAGGGCCCCAAGGGGCUGAGG

>miR-ex-71

GGUGGCCUGGGUGUAUGGUAGG

>miR-ex-72

AGGUCUCCAGCUUGGCCCUCCC

>miR-ex-73

AGUGGGCCGAGAUCGUGCCACU

>miR-ex-74

UGCCACUGCAUUCCAGCCUGGG

>miR-ex-75

UGUCCACACCCUCACCUCCAGA

>miR-ex-76

AGGAGGAGGAGGAGGGGCGGUG

>miR-ex-77

AGGCGGGAGAAUCGCUUGAACC

>miR-ex-78

CACUGCACUCCAGCCUGGGCGA

>miR-ex-79

UGAGGCAGGAGAAUUGCUUGAG

>miR-ex-80

UUGAGCCGGGAGGGUGGAGGCU

>miR-ex-81

UGAGGCUGGAGGAUGGCUUCAG

>miR-ex-82

CUUCAGCCUGGGAAGUUGAGGC

>miR-ex-83

CGGGAGGCUGAGGCAGGACAAU

>miR-ex-84

CACUGCACUCCAGCCUGGGCGA

>miR-ex-85

AGGCAGGAGACUUGCUUGAGCC

>miR-ex-86

CUUGAGCCCGGGAGGCGGAGGU

>miR-ex-87

UAGGGCGGCUGGCUGGGCGGGG

>miR-ex-88

CUCCCUCCCGGACGGGGCGGCU

>miR-ex-89

AGGCUGGAGUGCAGUGGCGUGA

>miR-ex-90

CACGCCAUUCCCCUGCCUCAGC

>miR-ex-91

ACAAUUCCCACGUGUCGUGGGA

>miR-ex-92

UUCUGUUCUAGUGAUAGUGGGU

>miR-ex-93

UCUCUCCGGGGUGGGGCGGUGG

>miR-ex-94

CAUCCCUGUCCCAGAUCCGGAA

>miR-ex-95

GUAGCCCCAGCUACUUGGGAGG

>miR-ex-96

UGAGGCAGGAGAAUGGCUUGAA

>miR-ex-97

UAAUCCCAGCUACUCGGGAGCC

>miR-ex-98

AACCCGGGAGGUGGAGGUUGCA

>miR-ex-99

UCCUGAGAGCUGGACUGCAGUG

>miR-ex-100

UUCUGGGUCUAGCUACCAGUGA

>miR-ex-101

GGCCUUCUCUGAUUGGACUUUA

>miR-ex-102

CAAAGUCCAAUCAGAGUAGGCC

>miR-ex-103

CUUGAACCCAGGAGGUGGAGGU

>miR-ex-104

GGCACUGUAAUCCCAGCUUCUU

>miR-ex-105

UAGGUAGGUGCAAAAGUAAUUG

>miR-ex-106

UCAAAAACUGCAGUUACUUUUG

>miR-ex-107

CUGCGAACGGAGUCCCCGCUGC

>miR-ex-108

UCCCCGCUGCCGCCUCUCAGCC

>miR-ex-109

CUGCGAACGGAGUCCCCGCUGC

>miR-ex-110

GUCCCCGCUGCCGCCUCUCAGC

>miR-ex-111

UGUGGGGUCCUUCCCCCGCCCC

>miR-ex-112

UCCCCUCCUCCCGCCCACGCCC

>miR-ex-113

UGUGGGGUCCUUCCCCCGCCCC

>miR-ex-114

CCCCCCCCCCCACGCCUCCUCC

>miR-ex-115

GGGGCGGCGCGCGCGCGCGCGC

>miR-ex-116

CGGCGGCGGCGGCGGGGGUGUG

>miR-ex-117

UGUGGGGUCCUCCCCCGCCCCC

>miR-ex-118

UCCCCUCCUCCCGCCCACGCCC

>miR-ex-119

GAGUGGACGGGAGCGGCGGGGG

>miR-ex-120

CGCGCGCGCGUGUGGUGUGCGU

>miR-ex-121

CGUCGGAGGGCGGCGGCGGCGG

>miR-ex-122

UCCCCUCCUCCCGCCCACGCCC

>miR-ex-123

GGGGCGGCGCGCGCGCGCGCGC

>miR-ex-124

CGCGCGUGUGGUGUGCGUCGGA

>miR-ex-125

UGAGCCGAGAUCUCGGCUGCAC

>miR-ex-126

UGCACUUCAGCCUGGGUGACAG

>miR-ex-127

CAGAAGGGUUGACACUGGGUUC

>miR-ex-128

GAGCUGCAAGUGCCAUGUGCUG

>miR-ex-129

AGUGGCACGAUCUCGGCUCACU

>miR-ex-130

CACUGCAGGCUCCGCCCCCCGG

>miR-ex-131

UCGGGGCGCGCUAGGCGGGGAG

>miR-ex-132

CGCGUGCGGCGCGAGCCGGGCG

>miR-ex-133

AGCUCCUCUGGGGGCUGAGGCA

>miR-ex-134

AACCUGGGAGGCAGAGGCUGCA

>miR-ex-135

GGCAGGAGAAUCAAUCAUUUGA

>miR-ex-136

CACUGCACUCCAGCCUGCAUGA

>miR-ex-137

CAGGGCAGGGGGAGUCCUCGUG

>miR-ex-138

UCGUGUCCCCUGCGCACAACAC

>miR-ex-139

GCGCCCAGGCUGGAGUGCAGUG

>miR-ex-140

ACUGCAACUUCCGCCUCCCGGG

>miR-ex-141

GGCCGCGCAAGGUGGAGUGAGC

>miR-ex-142

UCCCCACUCCCACCUGGCUCUA

>miR-ex-143

UCACCCGGGCUGGAGUGCAGUG

>miR-ex-144

ACUGCAAACUCCGCCUCUGGGG

>miR-ex-145

CCUUACCUCAGGUGAUCCAAAC

>miR-ex-146

UUGGGAUUACAGGGGUGAGCCA

>miR-ex-147

AUUCCUCUUCUCUGGGAAGGGC

>miR-ex-148

GCAUCUCUGAAAGAAAGGCAGC

>miR-ex-149

UGGGAAAGAGCAAAGAACCUGA

>miR-ex-150

UAAGGCACUCAGGUUCUUUGUU

>miR-ex-151

CUGCCCUUGUGAUCCACACCCG

>miR-ex-152

UGCUGGGAUUACAGGCGUGAGC

>miR-ex-153

UCAGCUUCCCAAAGUGCUGGGA

>miR-ex-154

CAGCACUUUGGGAGGCCAAGGU

>miR-ex-155

UCCCAGCUACUCAGGAGGCUGA

>miR-ex-156

UGAGGCAGGAGAAUCACUUGAA
