# Supplementary material for: Deciphering the Role of Putative Novel miRNAs Encoded From the Newly Found Genomic Regions of T2T‐CHM13 in the Progression of Collecting Duct Renal Cell Carcinoma
Source: Cancer Med. 2025 Apr 30;14(9):e70925. doi: 10.1002/cam4.70925 (PMC12042112; doi:10.1002/cam4.70925)
Supplement: Supplementary file 2 — Figure S1. KEGG pathway maps highlighting novel miRNA target genes downregulated in cdRCC, including, (A) Adipocytokine signaling pathway, (B) PPAR signaling pathway, (C) Apelin signaling pathway, (D) cGMP‐PKG signaling pathway, and (E) calcium signaling pathway. In each pathway map, novel miRNA target genes downregulated in cdRCC are highlighted with a yellow background. [file CAM4-14-e70925-s002.pdf]

Supplementary Figures

A)

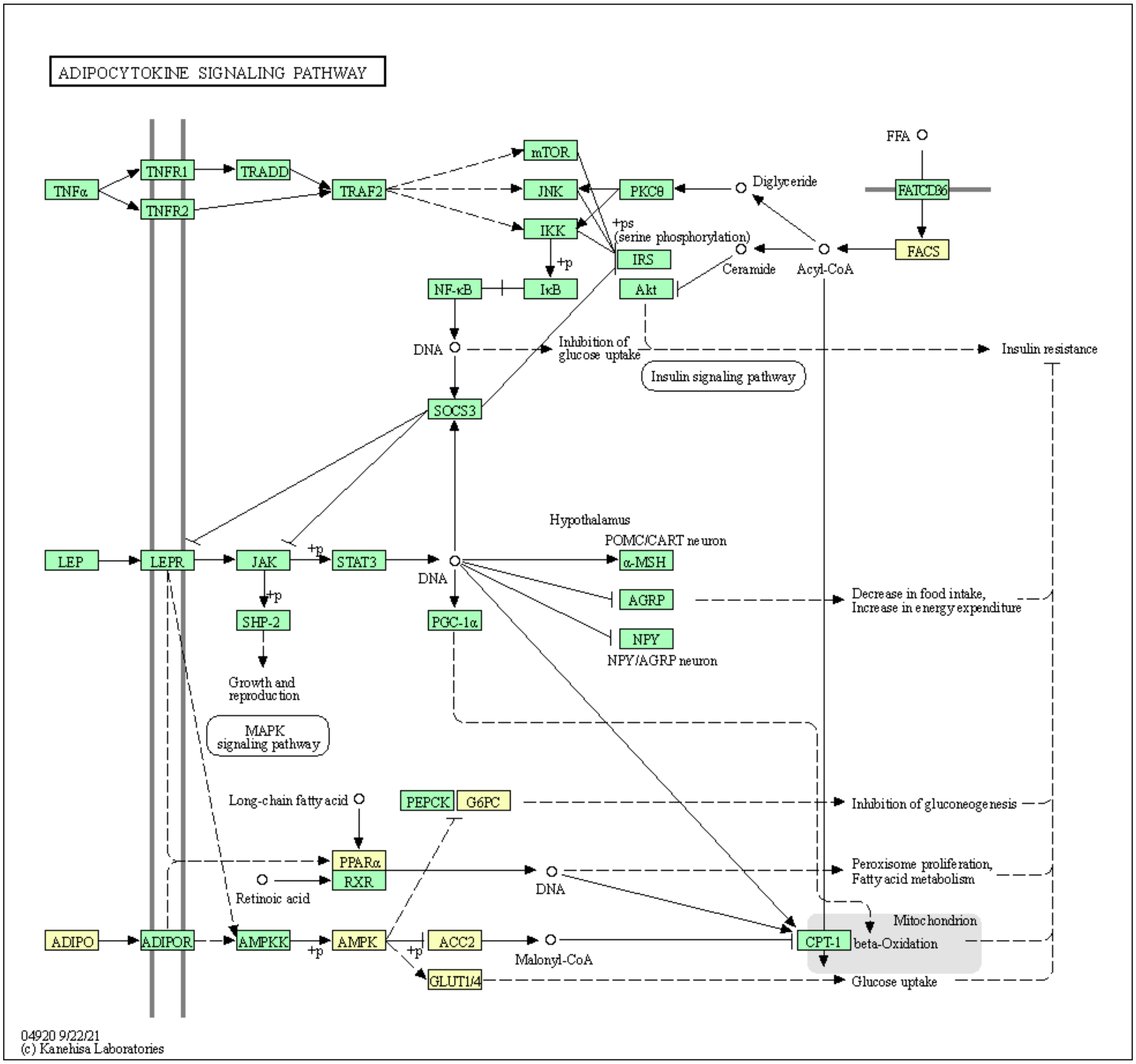

B)

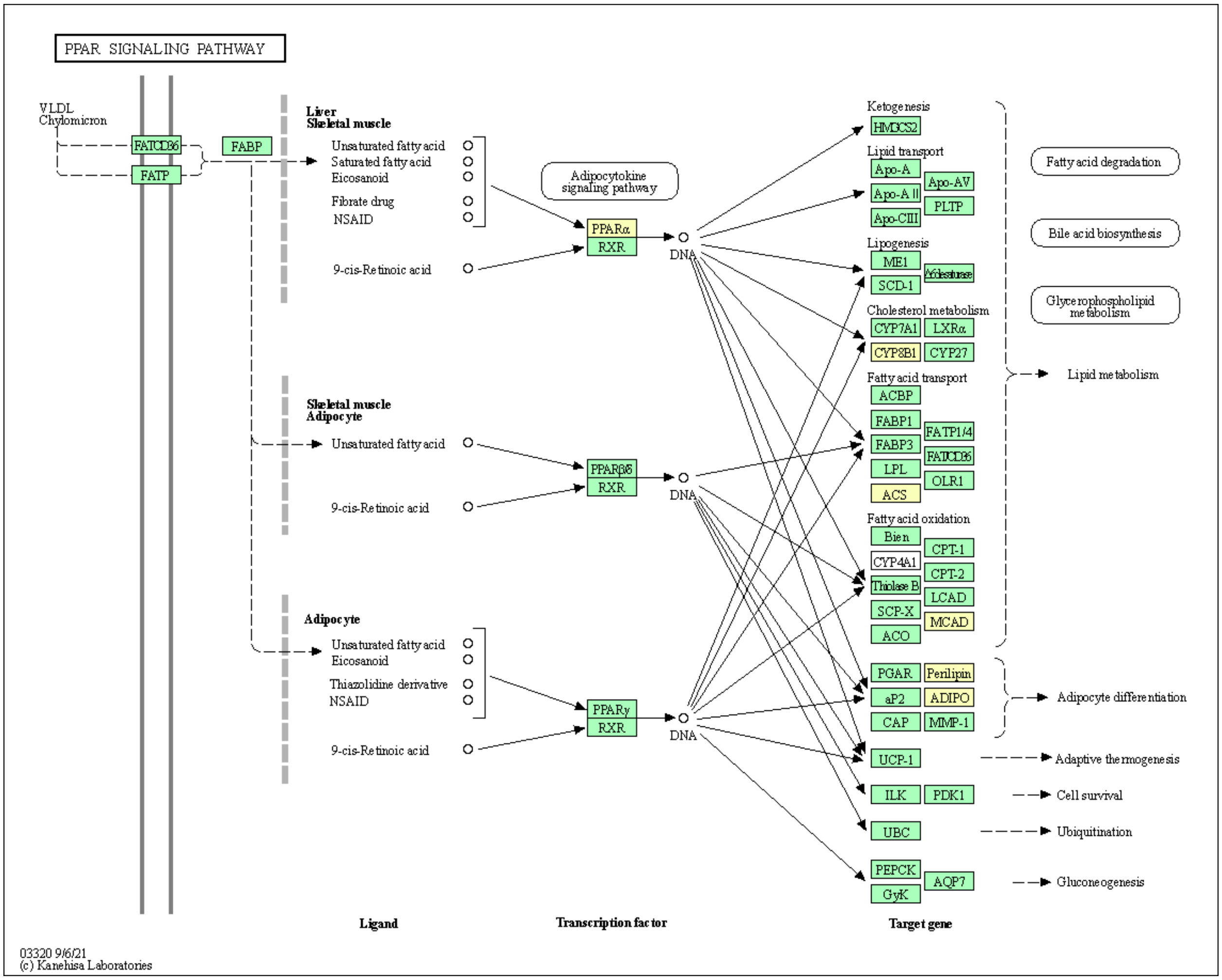
PPAR SIGNALING PATHWAY
VLDL
Chylomicron
FAT/CD36
FATP
FABP
Liver
Skeletal muscle
Unsaturated fatty acid
Saturated fatty acid
Eicosanoid
Fibrate drug
NSAID
9-cis-Retinoic acid
Adipocytokine signaling pathway
PPARα
RXR
DNA
Skeletal muscle
Adipocyte
Unsaturated fatty acid
9-cis-Retinoic acid
PPARβ/δ
RXR
DNA
Adipocyte
Unsaturated fatty acid
Eicosanoid
Thiazolidine derivative
NSAID
9-cis-Retinoic acid
PPARγ
RXR
DNA
Ketogenesis
HMGCS2
Lipid transport
Apo-A
Apo-AV
Apo-A II
PLTP
Apo-CIII
Lipogenesis
ME1
Δ9desaturase
SCD-1
Cholesterol metabolism
CYP7A1
LXRα
CYP8B1
CYP27
Fatty acid transport
ACBP
FABP1
FATP1/4
FABP3
FAT/CD36
LPL
OLR1
ACS
Fatty acid oxidation
Bien
CPT-1
CYP4A1
CPT-2
Thiolase B
LCAD
SCP-X
MCAD
ACO
PGAR
Perilipin
aP2
ADIPO
CAP
MMP-1
UCP-1
ILK
PDK1
UBC
PEPCK
AQP7
GyK
Fatty acid degradation
Bile acid biosynthesis
Glycerophospholipid metabolism
Lipid metabolism
Adipocyte differentiation
Adaptive thermogenesis
Cell survival
Ubiquitination
Gluconeogenesis
Ligand
Transcription factor
Target gene
03320 9/6/21
(c) Kanehisa Laboratories

C)

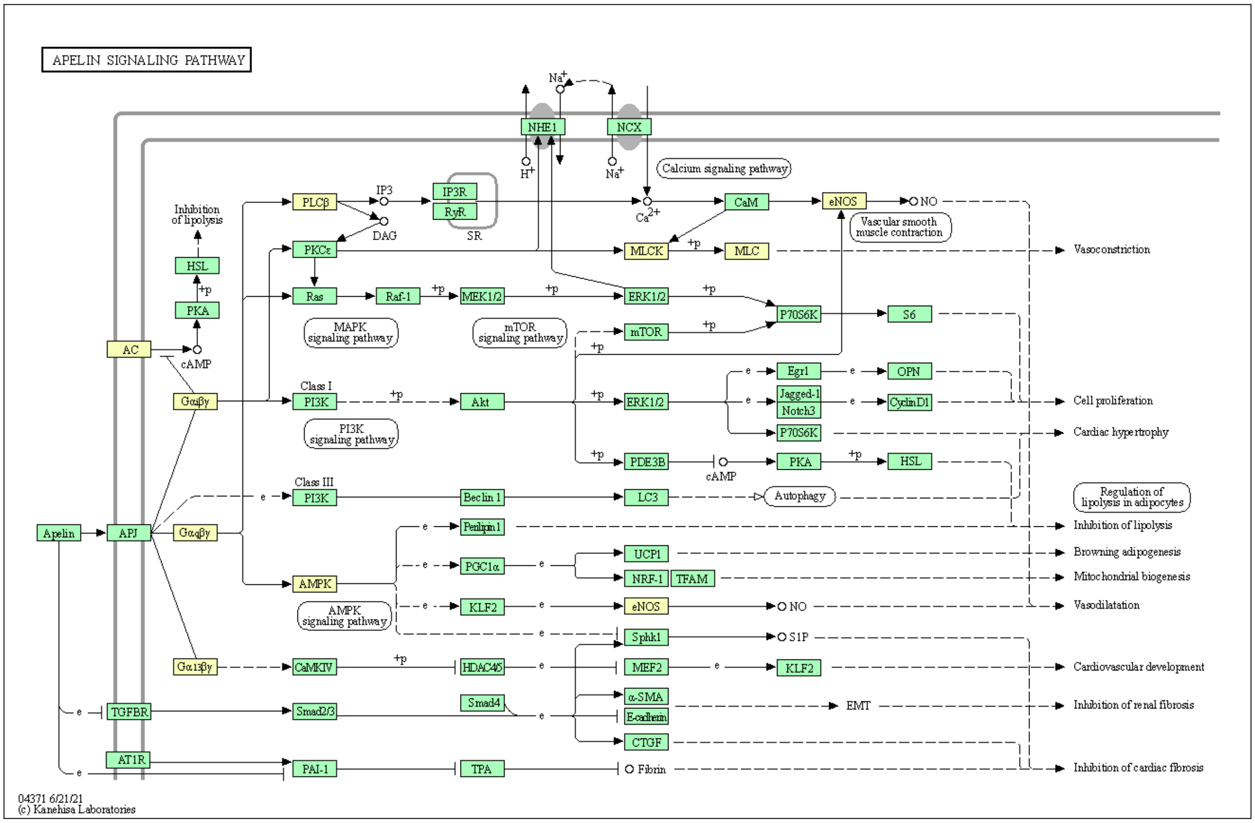
APELIN SIGNALING PATHWAY
Na+
NHE1
NCX
H+
Na+
Calcium signaling pathway
IP3
PLCβ
IP3R
RyR
SR
DAG
Ca2+
CaM
eNOS
NO
Vascular smooth muscle contraction
Inhibition of lipolysis
HSL
PKA
PKCε
MLCK
MLC
Vasoconstriction
Ras
Raf-1
MEK1/2
ERK1/2
P70S6K
S6
MAPK signaling pathway
mTOR signaling pathway
mTOR
AC
cAMP
Gαiβγ
Class I
PI3K
Akt
ERK1/2
Egr1
OPN
Jagged-1
Notch3
CyclinD1
Cell proliferation
P70S6K
Cardiac hypertrophy
PI3K signaling pathway
PDE3B
cAMP
PKA
HSL
Class III
PI3K
Beclin 1
LC3
Autophagy
Regulation of lipolysis in adipocytes
Apelin
APJ
Gαqβγ
Perilipin1
Inhibition of lipolysis
UCP1
Browning adipogenesis
AMPK
PGC1α
NRF-1
TFAM
Mitochondrial biogenesis
AMPK signaling pathway
KLF2
eNOS
NO
Vasodilatation
Sphk1
S1P
Gα13βγ
CaMKIV
HDAC4/5
MEF2
KLF2
Cardiovascular development
TGFBR
Smad2/3
Smad4
α-SMA
E-cadherin
EMT
Inhibition of renal fibrosis
CTGF
AT1R
PAI-1
TPA
Fibrin
Inhibition of cardiac fibrosis
04371 6/21/21
(c) Kanehisa Laboratories

D)

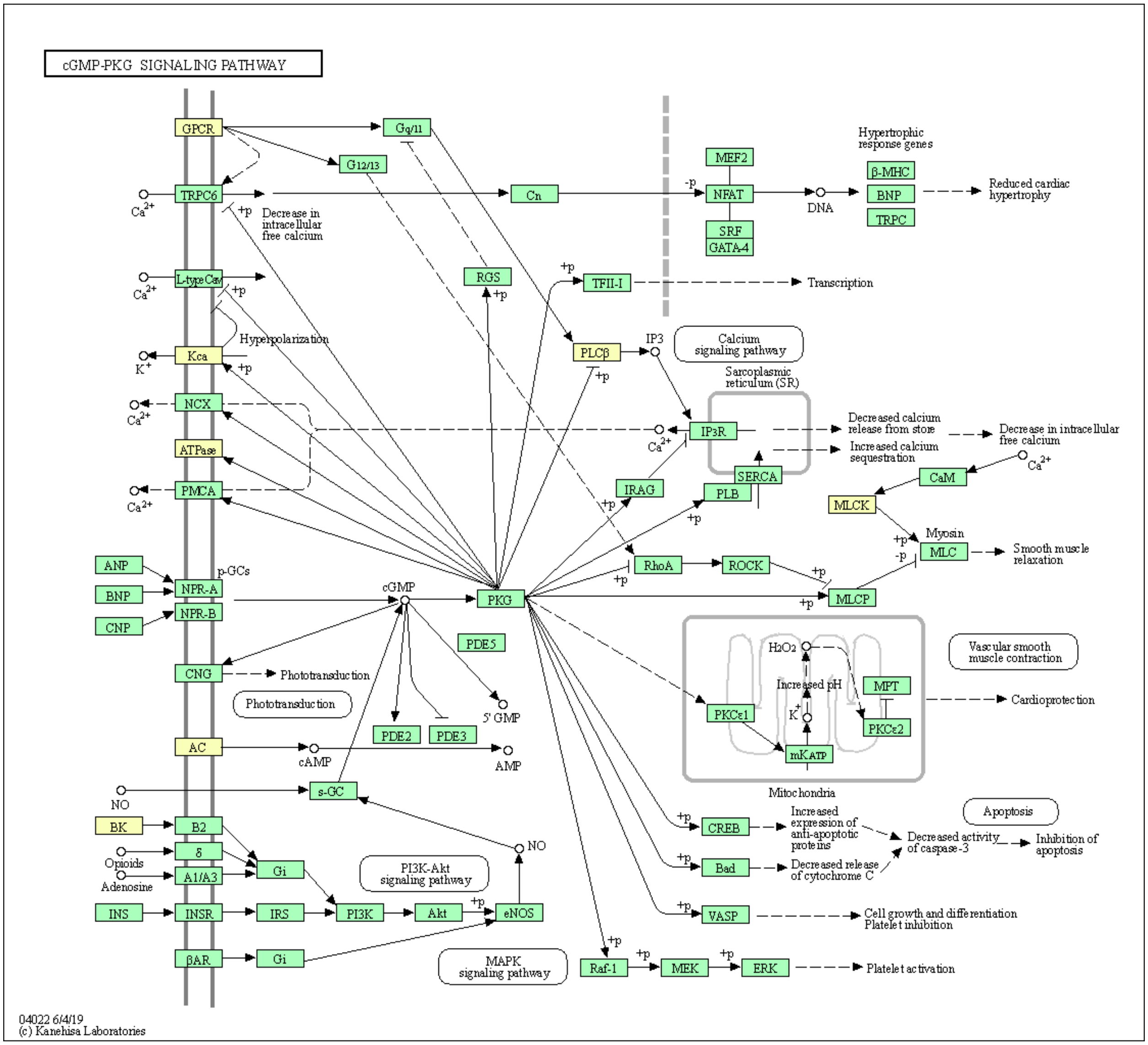

cGMP-PKG SIGNALING PATHWAY
GPCR
Gq/11
G12/13
TRPC6
Cn
MEF2
NFAT
SRF
GATA-4
DNA
Hypertrophic response genes
β-MHC
BNP
TRPC
Reduced cardiac hypertrophy
Decrease in intracellular free calcium
L-type Ca
RGS
TFII-I
Transcription
Kca
Hyperpolarization
PLCβ
IP3
Calcium signaling pathway
Sarcoplasmic reticulum (SR)
NCX
IP3R
Decreased calcium release from store
Increased calcium sequestration
Decrease in intracellular free calcium
ATPase
SERCA
CaM
PMCA
IRAG
PLB
MLCK
Myosin
MLC
Smooth muscle relaxation
ANP
BNP
CNP
NPR-A
NPR-B
p-GCs
cGMP
PKG
RhoA
ROCK
MLCP
PDE5
CNG
Phototransduction
H2O2
Increased pH
MPT
PKCε1
PKCε2
mKATP
Vascular smooth muscle contraction
Cardioprotection
Mitochondria
PDE2
PDE3
5' GMP
AC
cAMP
AMP
NO
s-GC
BK
B2
δ
Opioids
Adenosine
A1/A3
Gi
PI3K-Akt signaling pathway
INS
INSR
IRS
PI3K
Akt
eNOS
βAR
MAPK signaling pathway
CREB
Increased expression of anti-apoptotic proteins
Apoptosis
Decreased activity of caspase-3
Inhibition of apoptosis
Bad
Decreased release of cytochrome C
VASP
Cell growth and differentiation
Platelet inhibition
Raf-1
MEK
ERK
Platelet activation
04022 6/4/19
(c) Kanehisa Laboratories

E)

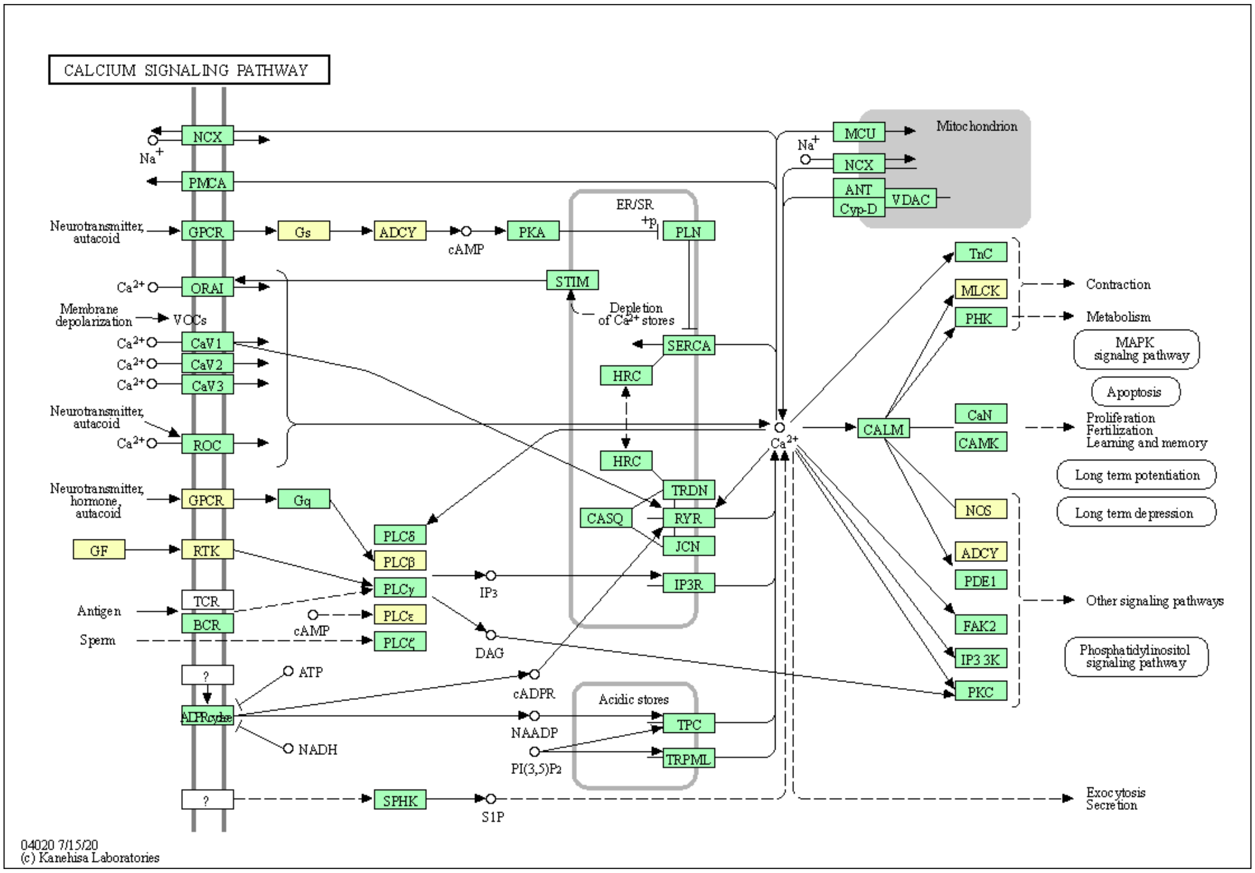

Figure S1: KEGG pathway maps highlighting novel miRNA target genes downregulated in cdRCC, including **(A)** Adipocytokine signaling pathway **(B)** PPAR signaling pathway **(C)** Apelin signaling pathway **(D)** cGMP-PKG signaling pathway, and **(E)** Calcium signaling pathway. In each pathway map, novel miRNA target genes downregulated in cdRCC are highlighted with a yellow background.
